# Antibacterial Activity and Untargeted Metabolomics Profiling of *Acalypha arvensis* Poepp

**DOI:** 10.3390/molecules28237882

**Published:** 2023-11-30

**Authors:** Valendy Thesnor, Roland Molinié, Ryland T. Giebelhaus, A. Paulina de la Mata Espinosa, James J. Harynuk, David Bénimélis, Bérénice Vanhoye, Catherine Dunyach-Rémy, Muriel Sylvestre, Yvens Cheremond, Patrick Meffre, Gerardo Cebrián-Torrejón, Zohra Benfodda

**Affiliations:** 1 UPR Chrome, University Nimes, CEDEX 1, 30021 Nîmes, France; valendy@gmail.com (V.T.); david.benimelis@unimes.fr (D.B.); patrick.meffre@unimes.fr (P.M.); 2 COVACHIM-M2E Laboratory EA 3592, Department of Chemistry, UFR SEN, Fouillole Campus, University of Antilles, CEDEX, 97110 Pointe-à-Pitre, France; muriel.sylvestre@univ-antilles.fr; 3 URE, Université d’État d’Haïti, Port-au-Prince HT6110, Haiti; yvens.cheremond@ueh.edu.ht; 4 UMR INRAE 1158 Transfrontalière BioEcoAgro, BIOlogie des Plantes et Innovation (BIOPI), UPJV, UFR de Pharmacie, 80037 Amiens, France; roland.molinie@u-picardie.fr (R.M.); berenice.vanhoye@u-picardie.fr (B.V.); 5 Department of Chemistry, University of Alberta, Edmonton, AB T6G 2N4, Canada; rgiebelh@ualberta.ca (R.T.G.); delamata@ualberta.ca (A.P.d.l.M.E.); harynuk@ualberta.ca (J.J.H.); 6 The Metabolomics Innovation Centre, Edmonton, AB T6G 2N4, Canada; 7 CHU Nîmes—Hôpital Universitaire Carémeau, CEDEX, 30900 Nîmes, France; catherine.remy@chu-nimes.fr

**Keywords:** *Acalypha arvensis* Poepp., antibacterial activity, SPME-GC×GC-TOFMS, untargeted metabolomics, Guadeloupe

## Abstract

The search for potent antimicrobial compounds is critical in the face of growing antibiotic resistance. This study explores *Acalypha arvensis* Poepp. (*A. arvensis*), a Caribbean plant traditionally used for disease treatment. The dried plant powder was subjected to successive extractions using different solvents: hexane (F1), dichloromethane (F2), methanol (F3), a 50:50 mixture of methanol and water (F4), and water (F5). Additionally, a parallel extraction was conducted using a 50:50 mixture of methanol and chloroform (F6). All the fractions were evaluated for their antimicrobial activity, and the F6 fraction was characterized using untargeted metabolomics using SPME-GC×GC-TOFMS. The extracts of *A. arvensis* F3, F4, and F5 showed antibacterial activity against *Staphylococcus aureus* ATCC 25923 (5 mg/mL), MRSA BA22038 (5 mg/mL), and *Pseudomonas aeruginosa* ATCC 27853 (10 mg/mL), and fraction F6 showed antibacterial activity against *Staphylococcus aureus* ATCC 29213 (2 mg/mL), *Escherichia coli* ATCC 25922 (20 mg/mL), *Pseudomonas aeruginosa* ATCC 27853 (10 mg/mL), *Enterococcus faecalis* ATCC 29212 (10 mg/mL), *Staphylococcus aureus* 024 (2 mg/mL), and *Staphylococcus aureus* 003 (2 mg/mL). Metabolomic analysis of F6 revealed 2861 peaks with 58 identified compounds through SPME and 3654 peaks with 29 identified compounds through derivatization. The compounds included methyl ester fatty acids, ethyl ester fatty acids, terpenes, ketones, sugars, amino acids, and fatty acids. This study represents the first exploration of *A. arvensis* metabolomics and its antimicrobial potential, providing valuable insights for plant classification, phytochemical research, and drug discovery.

## 1. Introduction

Antibiotics (ABs) are considered the most important medical discovery of the 20th century. ABs have revolutionized modern medicine and saved countless lives since their discovery. However, the overuse and misuse of antibiotics in human and animal health has led to the appearance of bacterial strains that have become resistant to most of the families of ABs available on the market [1,2,3,4]. In addition, the marketing of new antimicrobial molecules by the pharmaceutical industry has progressively decreased since the 1980s [5,6]. The World Health Organization (WHO) has warned that none of the ABs currently being developed would be enough to circumvent AB resistance [7]. Moreover, AB resistant infections are thought to be the cause of about 700,000 deaths annually, and by the year 2050 that number is expected to rise to 10 million [8]. Given this, there is an urgent need to identify new compounds with antibacterial properties that can be used to develop novel and effective ABs.

Currently, over 80% of pharmaceuticals on the market are of a natural product origin [9]. Therefore, medicinal plants are the target of investigations in drug discovery, as they are the main sources of bioactive phytochemicals [10,11]. Typically, traditional uses and knowledge of plants by indigenous peoples are studied to guide discoveries of novel medicinal plants [12]. Several studies have documented the traditional use of plants in the Caribbean to treat ailments such as coughs, respiratory issues, pain, and fever [12,13,14,15]. One such plant, *Acalypha arvensis* Poepp., a commonly used medicinal plant in the Caribbean, is being explored as a possible source of phytochemicals against infectious diseases associated with microorganisms [16].

*Acalypha arvensis* Poepp. (*A. arvensis*) is an annual or perennial plant belonging to the Euphorbiaceae family [17]. *A. arvensis* is typically 50 cm tall and features curved stems. *A. arvensis* is traditionally used by indigenous peoples of the Caribbean to treat a variety of ailments, including skin infections, gastrointestinal disorders, vaginitis, diarrhea, menstrual pain, and coughs [15,17,18]. Caceres et al. screened 50% ethanol extracts of *A. arvensis* against five human pathogenic enterobacteria (enteropathogenic *Escherichia coli*, *Salmonella enteritidis*, *Salmonella typhi*, *Shigelk dysenteriue*, and *Shigella flexneri*) and found the extract exhibited a zone of inhibition between 6 and 8 mm in diameter against *Salmonella typhi* and *Shigella flexneri* [18]. Additionally, a recent study by Ble-González et al. investigating an ethanoic extract from *A. arvensis* revealed its antibacterial activity against methicillin-sensitive and methicillin-resistant Staphylococcus aureus, Klebsiella pneumoniae, and Pseudomonas aeruginosa, along with the isolation of two bioactive compounds: corilagin and chlorogenic acid [19]. Furthermore, in another study, Caceres et al. demonstrated that *A. arvensis* showed a zone of inhibition greater than 8 mm in diameter against *Staphylococcus aureus*, a Gram-positive bacteria causing respiratory infections [20]. Despite the extensive bioassaying of *A. arvensis* extracts, few studies have performed untargeted metabolomic profiling on extracts of *A. arvensis*. Given this, we hypothesize that *A. arvensis* is a source of phytochemicals to fight against infectious diseases associated with microorganisms.

Here, we use untargeted metabolite profiling and bioassays to study the chemistry and bioactivity of *A. arvensis*. To our knowledge, this is the first comprehensive study to investigate the antibacterial activity, phytochemical composition (both volatile and non-volatile compounds), and metabolomic profile of *A. arvensis* from Guadeloupe. Metabolomics, which is the study of metabolite profiles in a biological system under a given set of conditions, has become a common approach to study plant chemistry and physiology [20,21,22,23]. Metabolomics has proven to be a valuable analytical tool for the identification of primary and secondary metabolites of medicinal plants, especially for evidence-based development of novel herbal and nutraceutical agents [21,22,23,24,25]. Untargeted metabolomics aims to thoroughly investigate the metabolome at the systems level and, therefore, attempts to profile all known and unknown metabolites in a plant extract [26]. The techniques used for metabolic profiling of plant extracts include nuclear magnetic resonance (NMR), gas chromatography–mass spectrometry (GC-MS), and liquid chromatography–mass spectrometry (LC-MS) [21,26]. Therefore, the first objective of this research was to study the antimicrobial activity of *A. arvensis* extracts collected in Guadeloupe. The second objective was to characterize the phytochemical and antimicrobial compounds of the plant extracts using untargeted metabolite profiling techniques, including comprehensive two-dimensional gas chromatography–time-of-flight mass spectrometry (GC×GC-TOFMS), GC-MS, and LC-MS. GC×GC-TOFMS metabolomic profiling was used to identify the volatile and non-volatile compounds of *A. arvensis* extracts using headspace solid-phase micro extraction (HS-SPME) and derivatization, respectively. HS-SPME/GC-MS in vitro analysis of the entire plant was undertaken. 

## 2. Results

### 2.1. Antibacterial Screening of Acalypha arvensis Poepp.

The antibiogram analysis included testing the F6 fraction, obtained using a mixture of methanol and chloroform, against various bacterial strains. At a concentration of 5 mg/mL, the extract effectively inhibited the growth of all bacterial strains tested, including four Gram-positive strains (*Staphylococcus aureus* ATCC 25923 (*S. aureus*), *Staphylococcus aureus* 024 (*S. aureus* 024), *Staphylococcus aureus* 003 (*S*. *aureus* 003), and *Enterococcus faecalis* ATCC 29212 (*E. faecalis*)) and two Gram-negative strains (*Escherichia coli* ATCC 25922 (*E*. *coli*) and *Pseudomonas aeruginosa* ATCC 27853 (*P. aeruginosa*)) (Table 1). Notably, the inhibition halos observed in the antibiogram were more pronounced for the susceptible strains, namely, *S*. *aureus* 003, *S*. *aureus* 024, and *S*. *aureus*. In addition to the antibiogram, all fractions were subjected to an overlay bioautography test to assess their antibacterial activity.

The screening revealed that the F3 (methanolic extract), F4 (methanol: water mixture extract), F5 (water extract), and F6 (methanol: chloroform mixture extract) fractions of *A. arvensis* exhibited antibacterial activity against both Gram-positive and Gram-negative bacterial strains (refer to ‘Bacterial Strains’ in the Materials and Methods section). Table 2 reports the inhibition of selected bacterial strains by these extracts, showing antibacterial activity against four out of the eight tested strains, methicillin-sensitive *S. aureus* ATCC 29213 (MS *S. aureus*), clinical resistant *S. aureus* MRSA BA22038 (MR *S. aureus*), *P. aeruginosa*, and *E*. *coli*.

Additionally, the fractions F3, F4, F5, and F6 were tested at lower concentrations (2 mg/mL–20 mg/mL) (Table 2). During the study, it was observed that the F6 fraction of *A. arvensis* exhibited significant inhibition effects against MS *S*. *aureus* and the resistant strain MR *S. aureus* at concentrations of 2 mg/mL and 5 mg/mL (Table 1 and Table 2). The F3 and F4 fractions also showed bacterial inhibition at 5 mg/mL against MS *S. aureus* and clinical MR *S. aureus*. Moreover, at a higher concentration of 10 mg/mL, the F3, F4, and F6 fractions exhibited an inhibitory effect not only on MS *S. aureus* and the resistant strain MR *S*. *aureus* but also on *P*. *aeruginosa*. Furthermore, F6 displayed an inhibitory effect on MS *S*. *aureus*, clinical MR *S*. *aureus*, *Enterococcus faecalis* ATCC 51299 (*E. faecalis* S), *P*. *aeruginosa*, and *E. Coli* at the highest tested concentration, which was 20 mg/mL.

### 2.2. Phytochemical Characteristics of A. arvensis

Chemical derivatization using specific visualizing reagents (refer to Phytochemical Screening in Materials and Methods section) was employed on TLC plates to determine the chemical composition of the compounds in *A. arvensis*. This technique is crucial for detecting colorless compounds that may not be visible under UV light or fluorescence. Universal reagents like vanillin sulfuric acid are commonly used in TLC to visualize a wide range of organic compounds. Additionally, selective reagents such as Neu’s reagent (2-aminoethyldiphenylboric acid + PEG) were utilized to identify flavonoids, while the Dragendorff reagent test was employed for alkaloid detection. In the case of *A. arvensis*, derivatization with Neu’s reagent yielded a positive reaction for compounds, indicating the presence of flavonoids, the Dragendorff reagent showed a positive result for compounds, suggesting the presence of alkaloids, and the Liebermann and Burchard reagent showed a positive result for terpenes, sterol, and steroids.

The phytochemical characteristics of the *A. arvensis* plant investigated are summarized in Table 3. The results show the presence of flavonoids, alkaloids, sugars, sterols, steroids and triterpenes, tannins, terpenoids, and saponins in *A. arvensis*. However, coumarins were not found in the plant. In order to identify the different components present in the various extracts of *A. arvensis*, various phytochemical tests were conducted. These tests were aimed at detecting the presence of specific chemical families or functions, such as flavonoids, alkaloids, sugars, sterols, steroids and triterpenes, tannins, coumarins, terpenoids, and saponins. The F1 to F6 fractions were prepared at concentrations of 5 mg/mL and applied onto a 20 × 20 cm silica gel Xtra SIL G UV254 TLC plate or placed into a test tube. In addition, specific chemical developers were also prepared to help reveal the presence of certain chemical families or functions.

### 2.3. Untargeted Analysis of GC×GC-TOFMS Metabolite Profiles of the Methanol: Chloroform Extract (50:50), F6

Untargeted derivatization and SPME-GC×GC-TOFMS metabolomics was performed on sample F6 of *Acalypha arvensis* (Poepp.), obtained from cold maceration of the dried and ground plant with a 50% methanol–chloroform mixture. This analysis allowed for the global exploration of all metabolites in *A. arvensis*, including both primary and secondary metabolites. The chromatograms were processed in ChromaTOF^®^ (v4.72), where peaks were detected and classified by their chemical family. These data are represented in the chromatograms (refer to Supplementary Materials).

This study specifically focused on analyzing the F6 fraction of *A*. *arvensis* using metabolomic techniques due to its higher inhibitory effects at a concentration of 2 mg/mL compared to the other fractions, which required higher concentrations for an inhibitory effect, ranging from 5 mg/mL to 20 mg/mL. Moreover, the maximum number of compounds was extracted by using the F6 fraction. The analysis involved derivatization and SPME-GC×GC-TOFMS, enabling the identification of both apolar and polar compounds present in the fraction. This comprehensive approach facilitated a detailed characterization of the metabolite composition within the F6 fraction of *A. arvensis*. 

**Figure 1:**
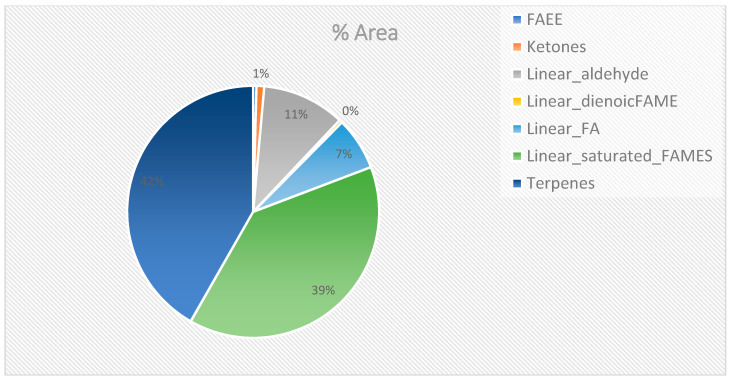
Compounds identified using SPME peaks that were detected by derivatization. Fatty acids (FAs); fatty acid ethyl esters (FAEEs); fatty acid methyl esters (FAMEs).

**Figure 2:**
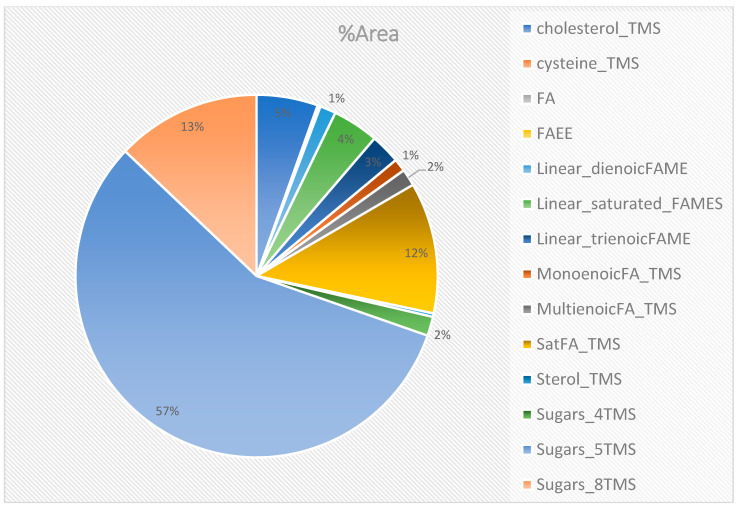
Compounds identified from derivatization peaks. Fatty acids (FAs); fatty acid ethyl esters (FAEEs); fatty acid methyl esters (FAMEs); trimethylsilyl ether (TMS); saturated fatty acid trimethylsilyl ether (SatFA_TMS).

The SPME-GC×GC-TOFMS analysis detected a total of 2861 peaks (Figure S8), including methyl ester fatty acids, ethyl ester fatty acids, terpenes, and ketones, while the derivatization analysis detected a total of 3654 peaks (Figure S8), including sugars, amino acids, and fatty acids. The identity of 29 compounds was confirmed for SPME (Figure 1), this corresponds to 42% terpenes, which are in the majority, followed by 39% linear saturated fatty acid methyl esters, 11% linear aldehydes, 7% linear fatty acids, and less than or equal to 1% ketones, linear dienoic fatty acid methyl esters, and fatty acid ethyl esters. Fifty-eight compounds were identified using derivatization, the majority, over 70%, were sugar trimethylsilyl ethers, 12% were saturated fatty acid trimethylsilyl ethers, and less than or equal to 5% were cholesterol-TMS, fatty acids, and fatty acid ethyl esters (Figure 2).

## 3. Discussion

In traditional medicine in the West Indies, *A. arvensis* is known for its antimicrobial properties. Therefore, the purpose of this study was to assess the antibacterial properties of *A. arvensis* and identify compounds present in the whole plant [27]. Previous research has shown that various species of the Acalypha genus possess antibacterial activity [28]. In vitro screening of *A. arvensis* extract, prepared with 50% ethanol, revealed its effectiveness against five human pathogenic enterobacteria, including enteropathogenic *E. coli*, *S. enteritidis*, *S. typhi*, *S. dysenteriae*, and *S. flexneri* [18]. More recently, the ethanolic extract of *A. arvensis* has demonstrated antibacterial activity against methicillin-susceptible and methicillin-resistant *S. aureus* 29213, *K. pneumoniae* 13883, and *P. aeruginosa* 27853 [19]. These findings align with our study, which showed that fractions F3, F4, F5, and F6 of *A. arvensis* exhibited antibacterial effects against MSSA, MRSA, *P*. *aeruginosa*, and *E. Coli*. Particularly, the F6 extract demonstrated inhibitory effects against *S. aureus*, *E. Coli*, *P*. *aeruginosa*, *E. faecalis*, *S. aureus* 024, and *S. aureus* 003. In our investigation, the *A. arvensis* extract has a halo of inhibition for SA of 13.67 ± 1.15 mm, higher than that of the *Acalypha mandonii* Müll. Arg. extract (11 mm against *S. aureus*) [29].

In general, the genus Acalypha is known to contain a variety of phytochemicals such as tannins, flavonoids, phenolic compounds, saponins, alkaloids, terpenoids, coumarins, anthocyanins, anthraquinones, and other bioactive compounds [28]. Our study aligns with these findings as we identified the presence of sugars, sterols, terpenoids, alkaloids, flavonoids, tannins, and saponins in the extracts of *A. arvensis*. Over the years, many of these compounds have demonstrated antibacterial activity [30,31,32,33,34].

The antibacterial activity observed in the F6 fraction can be attributed to the presence of various molecules identified through metabolomics analysis. This activity is likely linked to the presence of secondary metabolites, including terpenes, tannins, and flavonoids. Fraction F6 demonstrates a notable concentration of terpenes, suggesting a potential correlation between their presence and the observed antibacterial activity. Additionally, it is important to acknowledge that tannins and flavonoids are also present in our fraction. Notably, recent research [19] has unveiled the antibacterial properties of these compounds within the same plant (i.e., *A. arvensis*). Metabolomics studies have generally been presented as either non-targeted or targeted approaches. The non-targeted mode has been widely used in the fingerprinting of many medicinal and food plants. In contrast to targeted analyses, untargeted metabolomics allows for the discovery of as many metabolites as possible without necessarily identifying or quantifying a particular compound [35,36]. In our study, employing an untargeted GC×GC-TOFMS metabolomic analysis and HS-SPME/GC-MS analysis, we observed extensive variations in the chemical constituents of *A. arvensis* extracts, identifying a total of 143 different compounds out of the 145 compounds detected. Additionally, the antibacterial activity of sugar fatty acid esters was extensively studied, yielding variable results on different bacterial species. Some studies have reported inhibition of Gram-negative bacteria [37,38,39], while others have observed inhibition primarily against Gram-positive bacteria [39,40]. Recent studies have utilized techniques such as NMR and HPLC to identify eight specific compounds, including corilagin, chlorogenic acid, rutin, quercetin-3-O-glucoside, caffeic acid, treitol, (2R,3R)-butane-1,2,3,4-tetrol, (S,E)-1,3-Diphenylprop-2-en-1-ol, and (1R,2R,3R)-5-(hydroxymethyl)cyclohex-4-ene-1,2,3-triol, from the ethanolic extract of the aerial parts of *A. arvensis* in Mexico [19]. However, none of these compounds overlap with the ones we partially identified in our F6 extract. Therefore, metabolomic fingerprinting can serve as a valuable tool for identifying secondary metabolites and ensuring the quality assessment of medicinal plants [22]. The observed antibacterial activity in the F6 fraction can be attributed to a diverse array of molecules identified and not identified through the metabolomics analysis, encompassing both apolar and polar compounds. 

Terpenes are primarily hydrocarbons, but they encompass a broader category that includes structurally related derivatives like alcohols, aldehydes, ketones, and acids, known as terpenic compounds. These compounds are widely present in plants and often contribute to their characteristic scents. Terpenes are extracted from plants through methods such as steam distillation or cold pressing, resulting in the production of essential oils. In fact, terpenes constitute the most prevalent biochemical family found in essential oils.

Essential oils, which are produced by plants, are complex mixtures of various compounds, including phenols, aldehydes, ketones, alcohols, esters, and hydrocarbons. It is the combined presence of these compounds that gives essential oils their antimicrobial properties, among other potential benefits [41]. The effectiveness of essential oils can be influenced by the interactions between different terpenoid components within the oil. These interactions can lead to variations in the level of antimicrobial activity exhibited by the oil [42].

Tannins, on the other hand, have been found to inhibit the growth of both Gram-positive and Gram-negative bacteria, predominantly exhibiting a bacteriostatic effect rather than bactericidal activity [33]. This antibacterial activity is attributed to the structural properties of tannins, as they are macromolecular polyphenols rich in phenolic hydroxyl groups, which contribute to their potent antibacterial effects [33,43]. Among tannins, gallotannins have been shown to possess superior antibacterial efficacy compared to ellagitannins [44]. Tannic acid, a specific type of tannin composed of a central glucose unit and 10 galloyl groups, has been extensively studied for its broad-spectrum antibacterial activity and exhibits the lowest minimum inhibitory concentration (MIC) values [44]. 

Flavonoids, another class of compounds, have also garnered attention for their antibacterial properties. Numerous research groups have successfully isolated and identified flavonoids with antibacterial activity, and the antimicrobial potential of commercially available flavonoids has been quantified [31]. Examples of antibacterial flavonoids include apigenin, galangin, pinocembrin, ponciretin, genkwanin, sophoraflavanone G, as well as glycosides and derivatives of kaempferol [31].

Overall, the antibacterial activity observed in the F6 extract can be attributed to the collective presence and interactions of volatile compounds in essential oils, the structural properties of tannins, and the specific antibacterial flavonoids present in the extract.

## 4. Materials and Methods

### 4.1. Plant Material, Extraction, and Chemicals

Whole plants of *A. arvensis* were collected in Petit-Bourg, Guadeloupe (16°11′26″ N, 61°35′27″ W) from March 2021 to January 2022. One specimen was deposited at the herbarium of COVACHIM-M2E of Université des Antilles, Pointe-à-Pitre for identification and conservation (Voucher No. COVA22). Fresh plant material of *A. arvensis* was dried at room temperature for one week. The dried whole plant of *A. arvensis* was ground in a Spex planetary mill with a grinding bowl and tungsten carbide grinding balls. The extraction process involved a series of sequential Soxhlet extractions using solvents of increasing polarity (n-hexane, dichloromethane, methanol, methanol: water (50:50), and water). Additionally, a parallel maceration was performed using methanol: chloroform (50:50) as the solvent. Each extraction was carried out for 8 h. Through these extractions, we obtained distinct fractions: F1 (hexanoic extract), F2 (dichloromethane extract), F3 (methanolic extract), F4 (methanol: water mixture extract), and F5 (water extract). These fractions corresponded to the extracts obtained after using hexane, dichloromethane, methanol, methanol: water (50:50), and water solvents, respectively. Additionally, the F6 (methanol: chloroform mixture extract) fraction represented the fraction obtained from the extraction using methanol: chloroform (50:50). The dry extracts were carefully stored under cold conditions (−20 °C) until further analysis.

All analytical-grade solvents and liquid chromatography (LC)-MS-grade solvents used in this report were sourced from VWR (Radnor, PA, USA). High-performance liquid chromatography (HPLC) ultrapure water was prepared using a PURELAB Classic water purification system (Elga, Veolia), and pure analytical-grade formic acid was purchased from Sigma-Aldrich (St. Louis, MI, USA). All aluminum Si60 F 254 TLC plates (thickness 0.25 mm) were ordered from Merck (Darmstadt, Germany). For TLC chemical development, vanillin, 2-aminoethyl diphenylborinate, polyethylene glycol-4000 (PEG), and p-anisaldehyde were procured from Sigma-Aldrich, and glacial acetic acid and sulfuric acid were purchased from VWR. For TLC bioautography, Mueller–Hinton (MH) broth and MH agar were obtained from Carl Roth and Biokar Diagnostics, and INT was ordered from Sigma-Aldrich. The antibiotic 5 μg oxacillin was purchased from Bio-Rad, susceptibility disks code 66848, lot 4M5306. For derivatization, HPLC-grade methanol, HPLC-grade toluene, and 99.9% pyridine were purchased from Millipore-Sigma, Oakville, ON, Canada. The toluene was dried over anhydrous sodium sulfate (Millipore-Sigma, Oakville, ON, Canada). Methoxyamine hydrochloride (Millipore-Sigma, Oakville, ON, Canada) solution was prepared in pyridine at a concentration of 20 mg/mL. Ampoules of N-methyl-N-(trimethylsilyl) trifluoroacetamide + 1% trichloromethylsilane (MSTFA + 1% TMCS) were purchased from Fisher Scientific Canada and opened immediately prior to use. Safe-Lock amber centrifuge tubes were purchased from Eppendorf Canada Ltd., Mississauga, ON, Canada, while 2 mL glass GC vials, GC vials with integral 300 μL inserts, and GC vial caps (PTFE-faced silicon) were purchased from Chromatographic Specialities Inc. (Brockville, ON, Canada).

### 4.2. Bacterial Strains

The antibacterial effect of crude extracts of A. *arvensis* was initially assessed at the Institut Pasteur in Guadeloupe using an antibiogram method. The F6 (methanol: chloroform mixture) extract was tested against six bacterial strains, comprising four Gram-positive bacteria (*Staphylococcus aureus* ATCC 25923, *Enterococcus faecalis* ATCC 29212, *Staphylococcus aureus* 024, and *Staphylococcus aureus* 003 derived from pig and bovine nasal swabs) and two Gram-negative bacteria (*Escherichia coli* ATCC 25922 and *Pseudomonas aeruginosa* ATCC 27853). These bacterial strains were obtained from the collection of the Institut Pasteur in Guadeloupe, France.

Subsequently, at our laboratory at the Université de Nîmes, we evaluated the antibacterial effect of crude extracts of A. *arvensis* on various fractions: F1 (hexanoic extract), F2 (dichloromethane extract), F3 (methanolic extract), F4 (methanol: water mixture extract), F5 (water extract), and F6 (methanol: chloroform mixture extract). Bioautography on agar was employed against eight bacterial strains, encompassing three Gram-positive strains (*Staphylococcus aureus* ATCC 25923, clinical resistant MRSA BA 22038, and *Enterococcus faecalis* ATCC 31299) and five Gram-negative strains (*Klebsiella pneumonia* ATCC 700603, *Klebsiella pneumonia* clinical resistant BA 34029, *Pseudomonas aeruginosa* ATCC 27853, BMR clinical BA 35014, and *Escherichia coli* ATCC 25922). These bacterial strains were obtained from the microbiology service collection at CHU of Nîmes.

#### 4.2.1. Performance of the Antibiogram

In order to determine the sensitivity of various strains of bacteria to the extract, a Petri dish containing Muller–Hinton agar medium (MH) was used for each strain. The bacteria were plated on the agar, which was prepared from a culture in Tryptone Soy agar medium (TSA). A sample was taken from the agar medium (TSA) to create a suspension of 0.5 McFarland, which was used to inoculate the Petri dishes. Sterile paper discs were then placed on the dishes, and 10 µL of the sample (prepared with 5.5 mg of F6 (methanol: chloroform mixture extract) in 1 mL of DMSO) was added for imbibition. The dishes were then incubated for about 24 h at 37 °C, which is the optimal temperature for the growth of the studied bacteria. The sensitivity profile of the bacteria to the extract was determined by measuring the diameters of the inhibition zones around the discs on the dishes. The presence of translucent halos or inhibition zones, where the bacteria did not grow, and zones with no halo, where the bacteria proliferated, were observed. The measurement of the diameter of this halo gave us information about the effectiveness of the antibiotic. As there was no control to determine the sensitivity threshold, we considered a strain to be sensitive if the diameter of the inhibition zone was greater than 6 mm (diameter of the paper disc). Each experiment was performed in triplicate. For statistical analysis, the diameter values were measured and transferred to Excel (Microsoft Excel 2013, version 15.0.5363.1000).

#### 4.2.2. TLC Agar Overlay Bioautography

The bioautography was conducted following previous reports with minor modifications [45]. Bacterial suspensions: two colonies of each bacterial strain, listed above, were collected and placed in tubes containing 4 mL of the MH broth; then, the tubes were placed in the shaker incubator at 37 °C and 200 rpm overnight. After the bacteria were grown, twofold dilutions were performed for each bacterial strain (1 mL of the bacterial suspension in 9 mL of Ringer’s liquid) to obtain a concentration of 106 CFU/mL. Finally, 0.50 mL of the bacterial suspension (dilution 2) was then added to 15 mL of MH agar (kept liquid at 45 °C).

TLC plates: a first screening was performed with fractions F1, F2, F3, F4, F5, and F6 of *A. arvensis* to see which fractions presented antibacterial activity. For this, each crude extract was prepared at concentrations of 1 mg/mL, 5 mg/mL, 10 mg/mL, and 20 mg/mL and deposited, without migration, on the TLC plates. In addition, three antibiotics (cefotaxime, ofloxacime, and ampicillin), used in the treatment of infections caused by these bacteria, and a blank sample (methanol) were applied. 

The F3, F4, F5, and F6 fractions of *A. arvensis* showed activity against *Staphylococcus aureus* ATCC 25923 as well as clinical resistant SARM BA 22038 and *Pseudomonas aeruginosa* ATCC 27853, and were manually deposited using a Hamilton microsyringe (Bonaduz, Switzerland). Then, TLC plates were developed with ethyl acetate/methanol (90/10) eluent for the F3 and F6 fractions and hexane/ ethyl acetate (70/30) for the F4 eluent. This was not performed for the F5 fraction. After separation, the TLC plates were dried to completely remove the solvent. They were then sterilized under UV light and placed in Petri dishes. A volume of 15.5 mL of the agar–bacteria suspension was added to the Petri dish containing the TLC plate. After solidification of the medium, incubation was performed for 24 h at 37 °C. After incubation, a 2 mg/mL solution of INT was sprayed onto the surface of the agar–bacteria suspension and incubated again for 4 h. Inhibition was indicated by the presence of clear zones.

### 4.3. Phytochemical Screening

To identify the components of the various extracts of *A. arvensis*, phytochemical tests were conducted. These tests included the detection of flavonoids, alkaloids, sugars, sterols, steroids and triterpenes, tannins, coumarins, terpenoids, and saponins. The F1 to F6 fractions were prepared at a concentration of 5 mg/mL and applied onto a 20 × 20 cm silica gel Xtra SIL G UV254 TLC plate or placed into a test tube. To reveal the presence of certain chemical families or functions, specific chemical developers were also prepared.

1. To test for flavonoids, a Neu’s reagent or NP/PEG method was used. Two solutions were prepared for the test. Solution A, which was made up of 1 g of 2-aminoethyldiphenylboric acid and 100 mL of methanol, and solution B, which was made up of 5 g of PEG 4000 and 100 mL of ethanol. Then, a mixture of 10 mL of solution A and 8 mL of solution B was sprayed onto a TLC plate. The TLC plate was heated at 110 °C for approximately 2 min. The flavonoids were then observed under UV light at 366 nm and appeared as yellow, green, or orange fluorescent spots.
2. A Dragendorff reagent test was used to detect the presence of alkaloids. To perform the test, we first prepared two solutions. Solution A, which was made up of 0.85 g of basic bismuth nitrate and 10 g of tartaric acid dissolved in 40 mL of water, and solution B, which was made up of 16 g of KI dissolved in 40 mL of water. The two solutions were mixed extemporaneously with 5 mL of solution A, 5 mL of solution B, 100 mL of water, and 20 g of tartaric acid. The mixture was then sprayed onto a TLC plate. Alkaloids appeared as orange spots on the plate.
3. To detect the presence of sugars, a sulfuric thymol reagent test was used. A solution was prepared by dissolving 0.5 g of thymol in 95 mL of ethanol, and then 5 mL of concentrated sulfuric acid was added. The mixture was sprayed onto a TLC plate and heated at 110 °C for about 15 min. The sugars appeared as pink spots on the plate.
4. To identify the presence of sterols, steroids, and triterpenes, a Liebermann and Burchard reagent test was performed. A solution was prepared by mixing 5 mL of acetic anhydride, 5 mL of concentrated sulfuric acid, and 50 mL of 95% ethanol at low temperature just before use, then sprayed onto a TLC plate. The plate was then heated at 110 °C for 10 min. The compounds appeared as fluorescence at 366 nm under UV light.
5. A test was performed to detect the presence of tannins. A volume of 5 mL of extract was introduced into a test tube, and 0.5 mL of a 1% aqueous solution of FeCl_3_ was added. The presence of tannins was indicated by a greenish or blue-blackish color change in the solution.
6. A test was performed to identify the presence of coumarins. A volume of 5 mL of extract was placed in a test tube, to which 0.5 mL of a 10% solution of NH_4_OH was added. The mixture was then observed under UV light at 366 nm. An intense fluorescence indicated the presence of coumarins.
7. A test was conducted to detect the presence of terpenoids. A volume of 5 mL of extract was added to a mixture of 2 mL of chloroform and 3 mL of concentrated sulfuric acid. The presence of terpenoids was indicated by the formation of two layers and a brown color at the interface.
8. A foam test was performed to identify the presence of saponins. A volume of 10 mL of the extract was added to a test tube and shaken for a few seconds, then left to rest for 15 min. The presence of saponins was indicated by a persistent height of foam on top of the liquid.
9. To visualize the compounds using the sulfuric vanillin polyvalent developer, a solution was prepared by combining 1 g of vanillin, 2 mL of sulfuric acid, and 95% ethanol up to a total volume of 100 mL. After spraying the TLC plate with this solution, it was heated at 110 °C for around 5 min. The compounds present on the plate exhibited various colors depending on their chemical nature.

### 4.4. Isolation of Compounds from A. arvensis F6 Fraction Using SPME and Derivatization and GC×GC-TOFMS

#### 4.4.1. SPME Analysis

The dried plant material was transferred and accurately weighed (52.32 mg) into a 10 mL (Chromatographic Specialties, Brockville, ON, Canada) headspace vial. A Gerstel multipurpose autosampler was utilized for the SPME analysis. The sample underwent an incubation of 5 min at 60 °C, followed by an SPME extraction of 60 min at 60 °C. A desorption time of 3 min at 250 °C was used to introduce the sample into the GC×GC system. A Supelco^®^ (Supelco^®^, Bellefonte, PA, USA) StableflexTM CVB/CAR/PDMS SPME fiber was used. The analyses were performed using a Leco Pegasus 4D GC×GC-TOFMS (Leco Instruments, St. Joseph, MI, USA) with a cooled injection system (Gerstel, Linthicum, MD, USA), and liquid injection was using a multipurpose sampler (MPS) (Gerstel, Linthicum, MD, USA). The headspace vial was incubated for 10 min at 37 °C, followed by an SPME extraction of 60 min at 37 °C. A desorption time of 3 min at 250 °C was used to introduce the samples into the GC×GC system. A StableflexTM CVB/CAR/PDMS SPME fiber was used (Supelco, Bellefonte, PA, USA) [46].

The GC×GC-TOFMS system consisted of an Agilent 7890 (Agilent Technologies, Palo Alto, CA, USA) gas chromatograph and a Pegasus 4D TOFMS (LECO, St. Joseph, MI, USA) with a quad jet liquid-nitrogen-cooled thermal modulator. The first-dimension column was a 5% phenyl polysilphenylene-siloxane phase (Rtx^®^-5MS; 60 m × 0.25 mm i.d.; 0.25 μm film thickness) connected by means of a SilTiteTM μ-Union (Trajan Scientific and Medical, Victoria, Australia) to a second-dimension (2D) trifluoropropylmethyl polysiloxane-type phase (Rtx-200; 1.6 m × 0.25 mm i.d.; 0.25 μm film thickness). All columns were from Restek Corporation (Restek Corp., Bellefonte, PA, USA). The second-dimension column was installed in a separate oven located inside the main GC oven. The carrier gas was helium at a corrected constant flow rate of 2 mL/min, and the injector operated in solvent vent mode. The main oven temperature program was 40 °C (3 min hold), a ramp of 3.5 °C/min to 190 °C (no hold), and a final ramp of 15 °C/min to 290 °C (12 min hold). The secondary oven was programmed with a constant +5 °C offset relative to the primary oven. The modulation period was 2.50 s (0.40 s hot pulse and 0.85 s cold pulse time) with a +15 °C offset relative to the secondary oven. Mass spectra were acquired in the range *m*/*z* 40–800 at 200 spectra/s. The ion source temperature was set at 200 °C and the transfer line temperature was set at 240 °C. The detector voltage was run at an offset of −200 V relative to the tuning potential and the ionization electron energy (EI source) was set at −70 eV. Samples were acquired using the LECO ChromaTOF^®^ software, version 4.72.0.0. 

#### 4.4.2. Derivatization Analysis

A dried *A. arvensis* sample was transferred and accurately weighed (69.28 mg) in a 2 mL microcentrifuge tube (VWR, Radnor, PA, USA) and extracted in 1 mL 50:50 methanol (Optima Grade, Fisher Scientific, Hampton, NH, USA) and chloroform (HPLC-grade, Fisher Scientific). The extraction was vortexed for 5 min, then centrifuged at 10,000 rpm for 10 min (MIKRO 185, Hettich Zentrifugen, Westphalia, Germany). The supernatant was then removed and diluted by a factor of 40, yielding a concentration of 1.732 mg/mL, with 750 μL aliquoted into a 2 mL clear glass GC vial (Chromatographic Specialties) and capped. The extract was then dried under nitrogen at 40 °C. A 100 μL aliquot of toluene dried with anhydrous sodium sulfate was added to the sample and dried under nitrogen at 50 °C. A 50 μL aliquot of 20 mg/mL of methoxyamine hydrochloride (Fisher Scientific) in pyridine (HPLC Grade, Fisher Scientific) was added into the vial and incubated in the heating block for 1 h at 60 °C. Following this, a 75 μL aliquot of MSTFA + 1%TMCS (Fisher Scientific) was added to the vial and the sample was again incubated for 1 h at 40 °C. The sample was then transferred into a 300 μL glass insert autosampler vial (Chromatographic Specialties) and capped. 

#### 4.4.3. GC×GC-TOFMS Method

The analysis was performed using a LECO Pegasus 4D GC×GC-TOFMS (LECO Instruments, St. Joseph, MI, USA) with a cooled injection system (Gerstel, Linthicum Heights, MD, USA) and liquid injection was using a multipurpose sampler (MPS) (Gerstel, Linthicum Heights, MD, USA). The first-dimension column was a 60 m × 0.25 mm × 0.25 μm Rxi-5SilMS, and the second-dimension was a 1.2 m × 0.25 mm × 0.25 μm Rtx-200MS (Chromatographic Specialties). Ultra-pure helium (5.0 grade; Praxair Canada Inc., Edmonton, AB, Canada) was used as the carrier gas, with a constant flow rate of 2.0 mL/min. The injection was splitless, using a split liner (Gerstel, Linthicum Heights, MD, USA) and an injection volume of 1 μL. The inlet temperature started at 80 °C and was then ramped to 250 °C within a minute for all runs. The temperature program of the primary oven began at 80 °C, held for 4 min, followed by a ramp of 3.5 °C/min until a temperature of 315 °C, which was held for 10 min. The secondary oven and modulator temperature offset were constant at +10 °C and +15 °C, respectively. The modulation period was 2.5 s. Mass spectra were collected at an acquisition rate of 200 Hz over a mass range between 40 and 800 *m*/*z.* The detector voltage was 1700 V with an electron impact energy of −70 eV. The ion source temperature was 200 °C with a transfer line temperature of 250 °C.

#### 4.4.4. Data Processing and Analysis

The GC×GC-TOFMS data were processed using ChromaTOF^®^ (v.4.72; LECO). The baseline offset was set to 0.9 above the middle of the noise. The minimum S/N ratio for the base and sub-peaks was set at 50, and the mass spectral match required for the sub-peaks to be included in the auto-smoothed peak was set at 650. The expected peak widths throughout the entire chromatographic run were assumed to be approximately 10 s in the first dimension and 0.15 s in the second dimension. Library matching for putative compound identification was performed against commercially available and in-house databases. Regions of the chromatogram containing siloxanes and derivatization reagents were excised and data from these regions was not included in the peak tables.

The resulting chromatograms were processed with automated filtering scripts and displayed as a contour map [47]. The color scale on the map indicated the intensity or concentration of the peaks, with red denoting high intensity and dark blue representing the baseline or background (refer to part 2 in Supplementary Materials). Each peak on the map corresponded to a single compound on the two-dimensional plot.

## 5. Conclusions

The chemical composition analysis of *A. arvensis* in this study revealed its antibacterial activity against Gram-positive and Gram-negative bacteria. Non-targeted derivatization and SPME-GC×GC-TOFMS metabolomics detected a total of 2861 peaks using SPME and 3654 peaks using derivatization. Phytochemical screening further identified sugars, sterols, terpenoids, alkaloids, flavonoids, tannins, and saponins in the extracts of *A. arvensis*. These findings highlight the value of untargeted metabolomics in studying plant metabolomes. The established methodology using advanced GC×GC-TOFMS techniques enabled a comprehensive examination of the chemical composition of *A. arvensis*. This understanding of the metabolite distribution in the plant can contribute to classification, authentication, future phytochemical research, and potential therapeutic applications. Integrating this metabolomic data with an assessment of their biological activities could facilitate a more targeted isolation process and enhance the efficiency of drug discovery efforts.

## Figures and Tables

**Table 1:** Antibiogram test with the diameter of the zone of inhibition (mm).

	Gram-Positive Bacteria				Gram-Negative Bacteria	
	*Staphylococcus aureus* ATCC 25923	*Enterococcus faecalis* ATCC 29212	*Staphylococcus aureus* 024	*Staphylococcus aureus* 003	*Pseudomona aeruginosa* ATCC 27853	*Escherichia coli* ATCC 25922
F6	5.5 mg/mL					
Diameter (mm)	13.67 ± 1.15	12.5 ± 0.71	13 ± 0	14 ± 0	7 ± 0	13 ± 0
Oxa5	5 µg					
Diameter (mm)	46 ± 1	N/D	46 ± 1	36 ± 1	N/D	N/D

Antibacterial activity of the F6 (5.5 mg/mL) sample against the test bacterial strains. Data are expressed as mean ± SD, where n = 3. Oxacillin 5 µg: Oxa5. N/D: non-determined.

**Table 2:** Bioautography results on TLC plate.

	Gram-Positive Bacteria			Gram-Negative Bacteria				
Fractions	*S. aureus* ATCC 29213	Clinical SARM BA 22038	*E. faecalis* ATCC 51299	*K. pneumonia* ATCC 700603	Clinical resistant *K. pneumonia* BA 34029	*P. aeruginosa* ATCC 27853	Clinical BMR BA 35014	*E. coli* ATCC 25922
	2 mg/mL							
F6	V	V	X	X	X	X	X	X
Fraction	5 mg/mL							
F3	V	V	X	X	X	X	X	X
F4	V	V	X	X	X	X	X	X
F6	V	V	X	X	X	X	X	X
Fraction	10 mg/mL							
F3	V	V	X	X	X	V	X	X
F4	V	V	X	X	X	V	X	X
F6	V	V	V	X	X	V	X	X
Fraction	20 mg/mL							
F3	V	V	X	X	X	V	X	V
F4	V	V	X	X	X	V	X	V
F5	V	V	X	X	X	V	X	X
F6	V	V	V	X	X	V	X	V

For all strains, positive controls were tested with ketofaxime 1 mg/mL, ofloxacime 1 mg/mL, ampicillin 1 mg/mL, and a negative control, the solvent of dissolution. V: inhibition effect; X: no inhibition effect.

**Table 3:** Qualitative phytochemical screening of A. *arvensis* fractions.

	Flavonoids (Neu’s Reagent)	Alkaloids (Dragendorff Reagent)	Sugars (Sulfuric Thymol Reagent)	Sterols, Steroids, and Triterpenes (Liebermann and Burchard Reagent)	Tannins	Coumarins	Terpenoids	Saponins
F1	−	−	−	+	−	−	+	−
F2	−	−	−	+	−	−	−	−
F3	+	+	+	+	+	−	−	−
F4	+	+	+	+	+	−	−	−
F5	+	−	+	−	+	−	−	+
F6	+	+	+	+	+	−	+	−

+: Presence of compound; −: absence of compound.

## Data Availability

Data are contained within the article.

## Supplementary Materials

The following supporting information can be downloaded at: https://www.mdpi.com/article/10.3390/molecules28237882/s1. Figure S1: Photograph of *Acalypha arvensis* Poepp individual; Figure S2: GC×GC total ion chromatogram *A. arvensis* extract F6 derivatized; Figure S3: GC×GC total ion chromatogram *A. arvensis* extract F6 derivatized (with peak markers); Figure S4: GC×GC total ion chromatogram *A. arvensis* extract F6 derivatized with scripts; Figure S5: GC×GC total ion chromatogram *A. arvensis* extract F6 SPME; Figure S6: GC×GC total ion chromatogram *A. arvensis* extract F6 SPME (with peak markers); Figure S7: GC×GC total ion chromatogram *A. arvensis* extract F6 SPME with Scripts; Figure S8: GC×GC methods comparison; Table S1. Fifty-eight compounds identified by derivatization peaks that were detected by derivatization. 1tR and 2tR represent primary and secondary retention times, respectively; Table S2. Twenty-nine compounds identified by SPME peaks that were detected by derivatization. 1tR and 2tR represent primary and secondary retention times, respectively; Figure S9. TLC plate of all fractions of Acalypha arvensis Poepp.
