# Supplementary material for: Antibacterial Activity and Untargeted Metabolomics Profiling of Acalypha arvensis Poepp
Source: Molecules. 2023 Nov 30;28(23):7882. doi: 10.3390/molecules28237882 (PMC10708339; doi:10.3390/molecules28237882)
Supplement: Supplementary file 1 [file molecules-28-07882-s001.zip › molecules-2670324-supplementary.pdf]

Supporting Information

Antibacterial activity and untargeted metabolomics profiling of *Acalypha arvensis* Poepp

Valendy Thesnor[1,2,3], Roland Molinié[4], Ryland T. Giebelhaus[5,6], A. Paulina de la Mata Espinosa[5,6], James J. Harynuk[5,6], David Bénimélis[1], Bérénice Vanhoye[4], Catherine Dunyach-Rémy[7], Muriel Sylvestre[2], Yvens Cheremond[3], Patrick Meffre[1], Gerardo Cebrián-Torrejón[2,8*] and Zohra Benfodda[1,8*]

[1] UNIV. NIMES, UPR CHROME, F-30021 Nîmes cedex 1, France.
[2] COVACHIM-M2E Laboratory EA 3592, Université des Antilles, Fouillole Campus, UFR SEN, Department of Chemistry, Pointe-à-Pitre Cedex, France.
[3] URE, Université d'État d'Haïti, Port-au-Prince, Haïti.
[4] UMR INRAE 1158 Transfrontalière BioEcoAgro, BIOlogie des Plantes et Innovation (BIOPI), UPJV, UFR de Pharmacie, 80037 Amiens, France
[5] Department of Chemistry, University of Alberta, Edmonton, AB T6G 2N4, Canada
[6] The Metabolomics Innovation Centre, Edmonton, AB T6G 2N4, Canada
[7] CHU Nîmes - Hôpital Universitaire Carémeau, Nîmes cedex, France.
[8] The authors supervised joinly this work
* Correspondence: G.C.T.: gerardo.cebrian-torrejon@univ-antilles.fr and ZB: zohra.benfodda@unimes.fr

Table of contents

1. Photograph of *Acalypha arvensis* Poepp. Individual
2. Figures untargeted analysis of GC × GC-TOFMS metabolite profiles of the methanol: chloroform extract (50:50), F6
3. Tables untargeted analysis of GC × GC-TOFMS metabolite profiles of the methanol: chloroform extract (50:50), F6
4. TLC plate of all fraction of *Acalypha arvensis* Poepp

1. Photograph of *Acalypha arvensis* Poepp. Individual

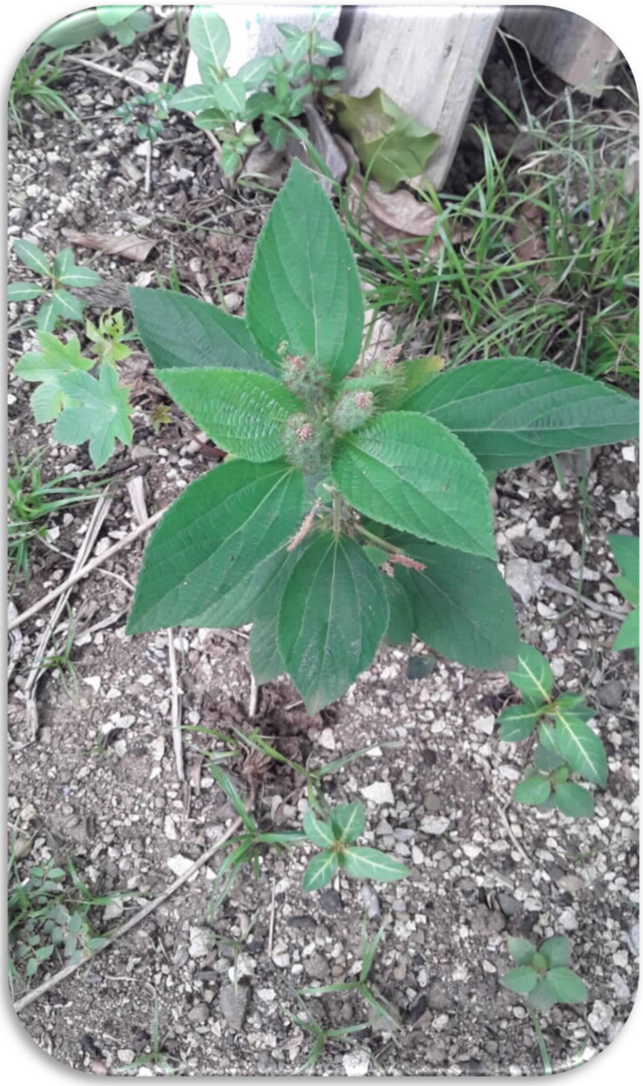

Figure S1: Photograph of a *Acalypha arvensis* Poepp (taken by Josia Richardson)

2. Figures untargeted analysis of GC × GC-TOFMS metabolite profiles of the methanol: chloroform extract (50:50), F6

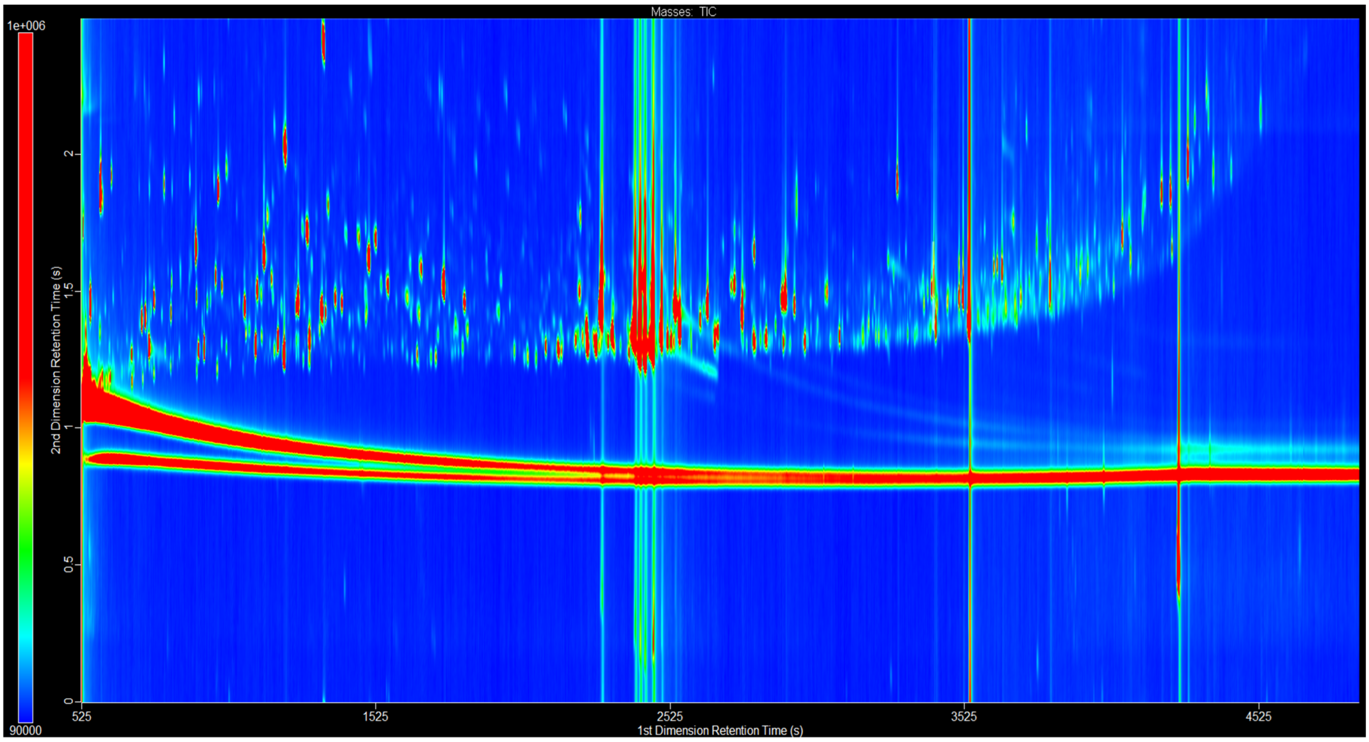

Figure S2: GC×GC total ion chromatogram A. *arvensis* extract F6 derivatized

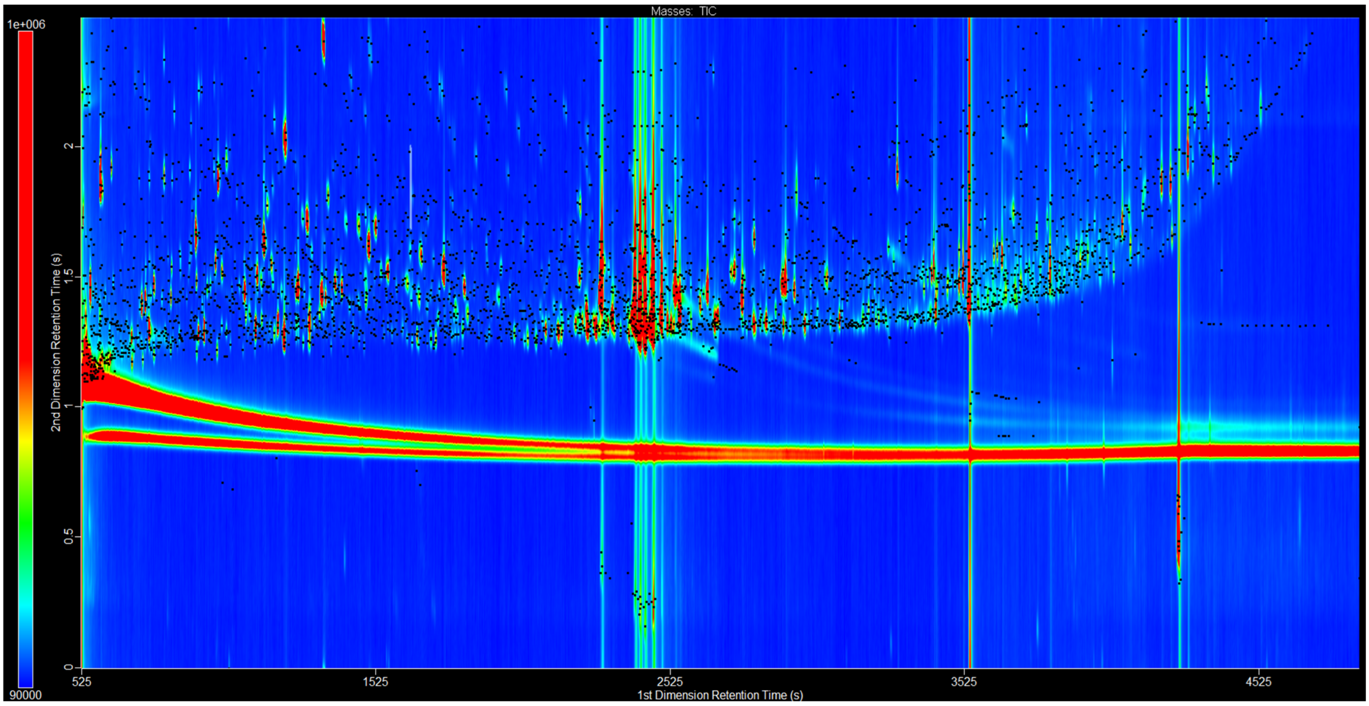

Figure S3: GC×GC total ion chromatogram A. arvensis extract F6 derivatized (with peak markers)

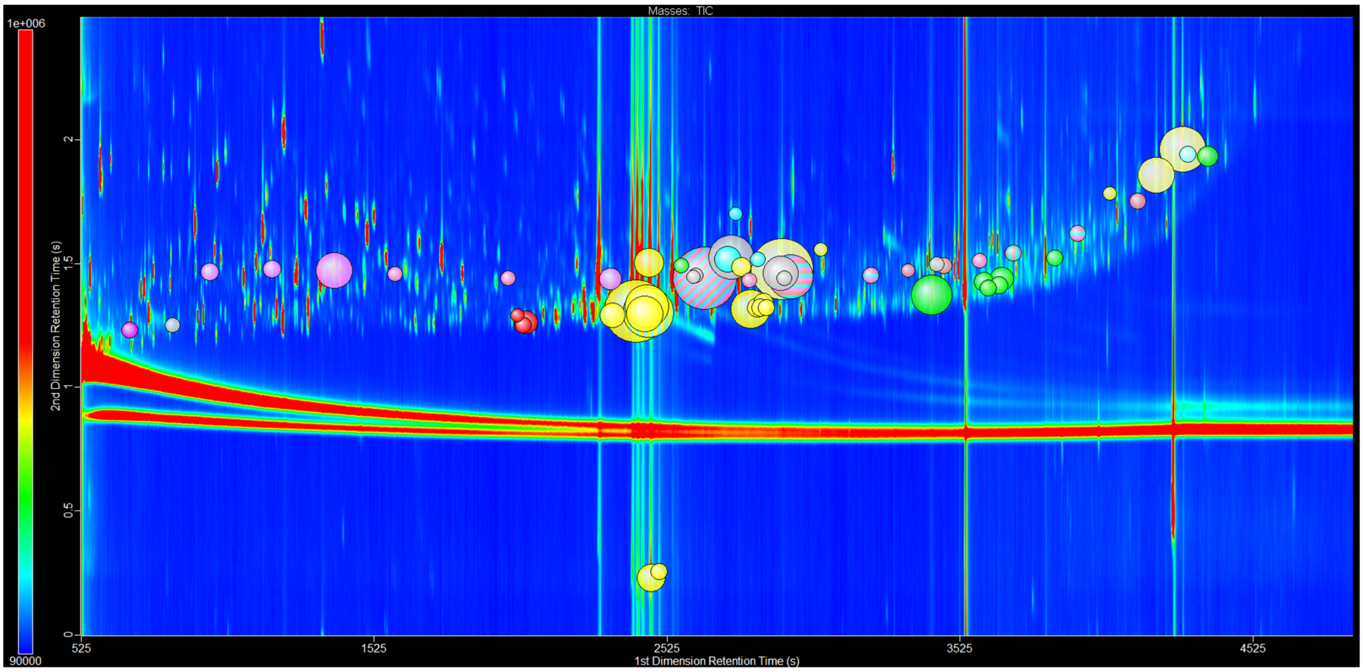

Figure S4: GC×GC total ion chromatogram A. *arvensis* extract F6 derivatized with Scripts

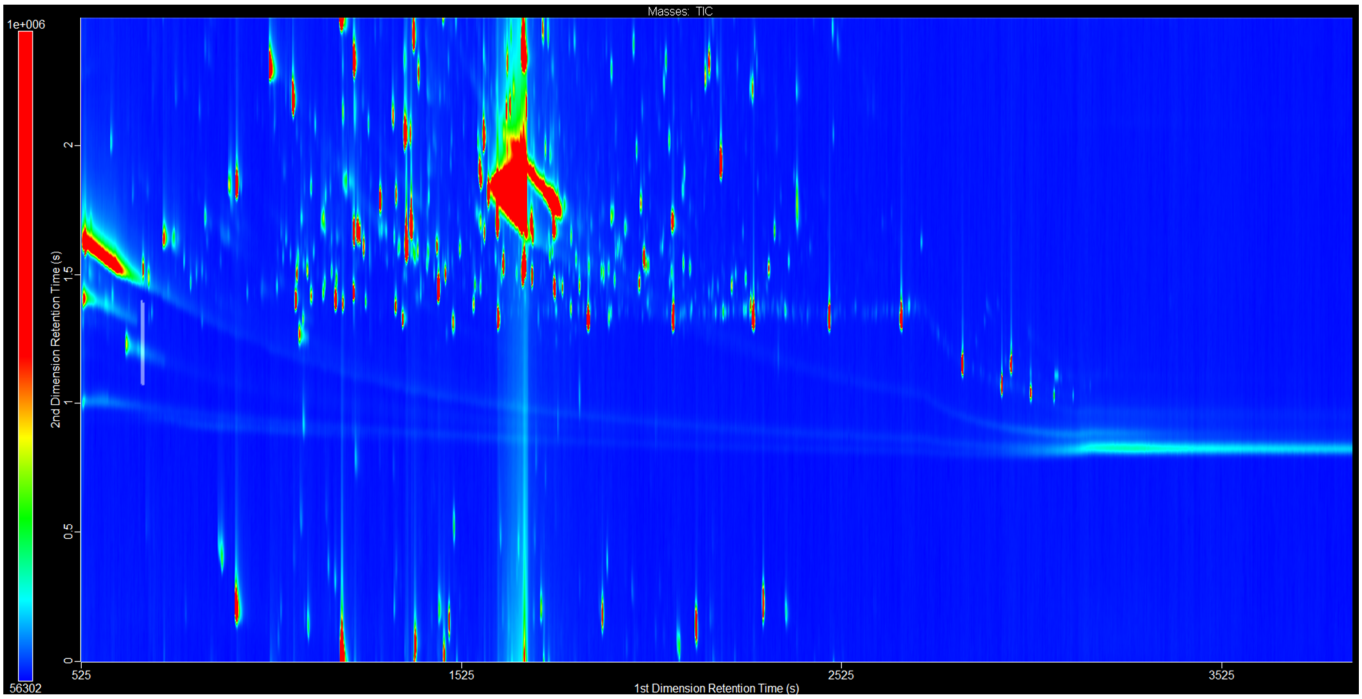

Figure S5 : GC×GC total ion chromatogram A. *arvensis* extract F6 SPME

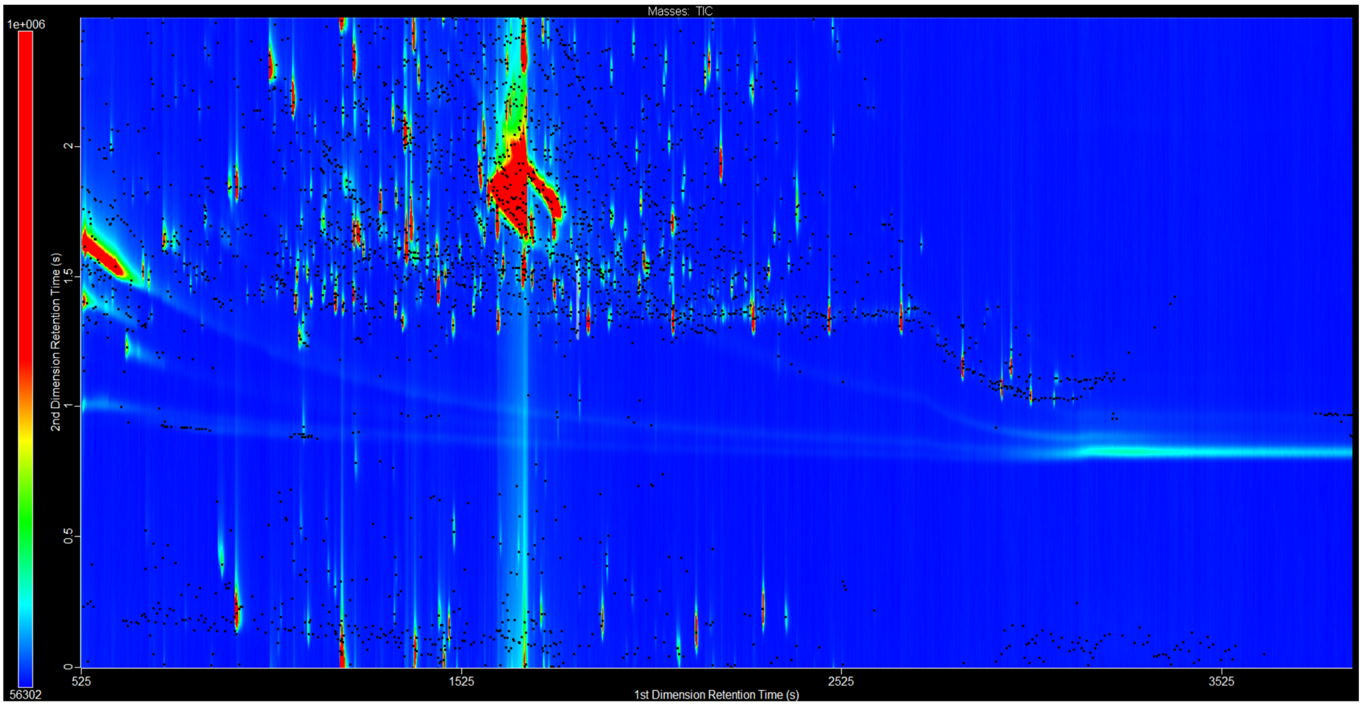

Figure S6: GC×GC total ion chromatogram A. *arvensis* extract F6 SPME (with peak markers)

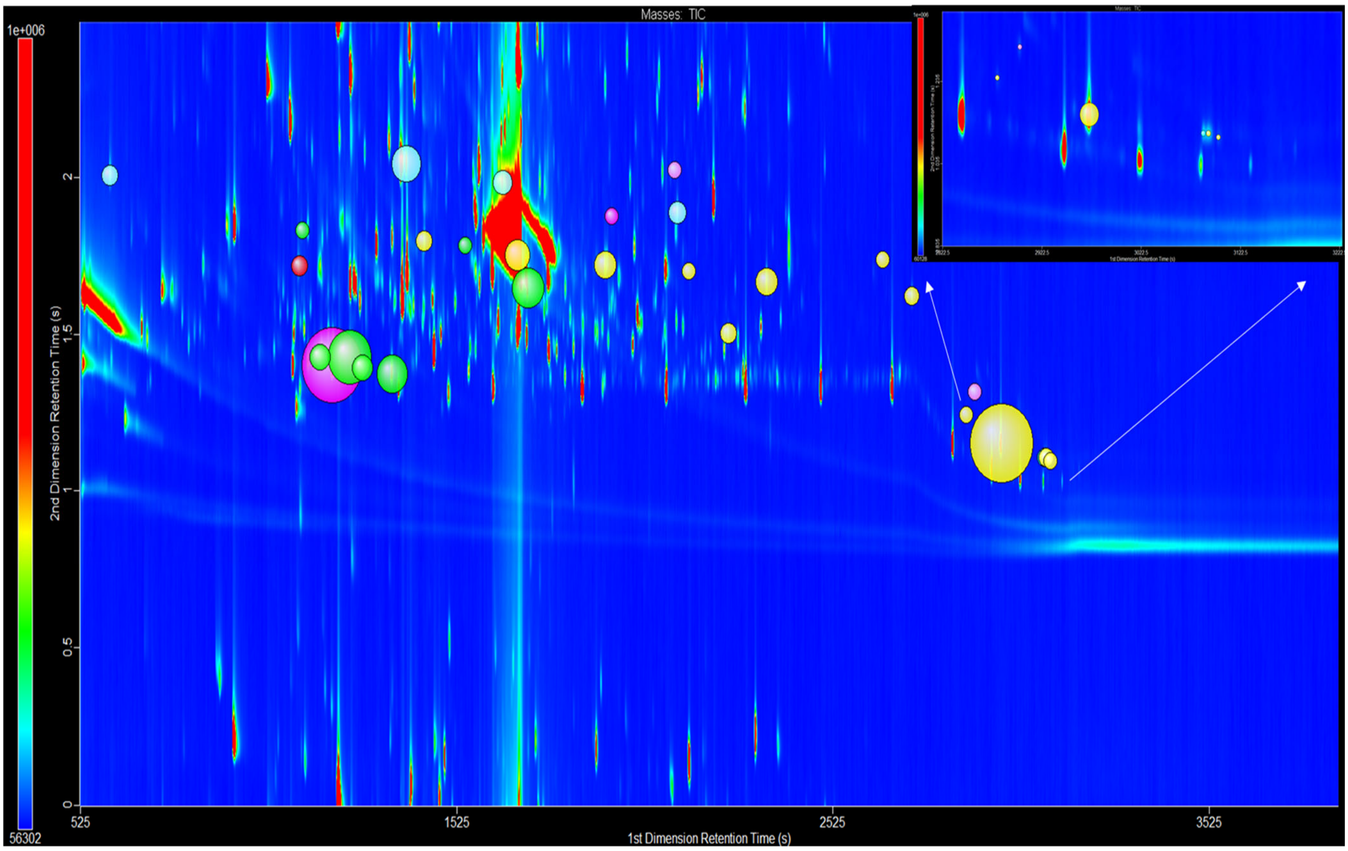

Figure S7: GC×GC total ion chromatogram A. *arvensis* extract F6 SPME with Scripts

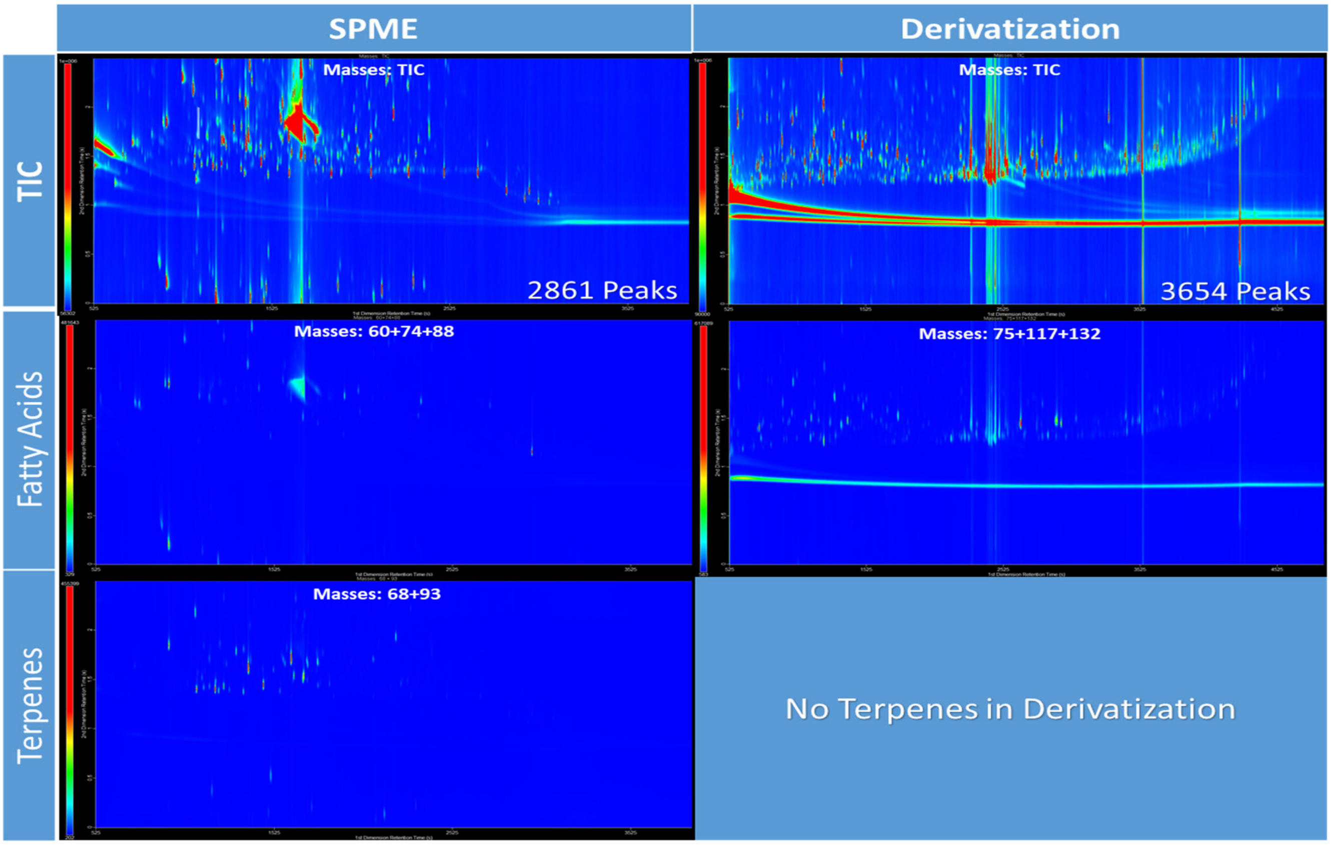

Figure S8 : GC×GC Methods Comparison

3. Tables untargeted analysis of GC × GC-TOFMS metabolite profiles

Table S1. Fifty-eight compounds identified by derivatization peaks that were detected by derivatization. ^{1}tR, ^{2}tR represent primary and secondary retention times, respectively.

Peak #	Name	Similarity	Classifications	^{1}tR, ^{2}tR (s)	UniqueMass	Quant S/N	Area
417	Limonene	936	monoterpene	687.5 , 1.235	68	1707	283112
647	Tris(trimethylsilyl)hydroxylamine	680	cysteine_TMS	835 , 1.255	146	410.06	37014
841	Heptanoic acid, trimethylsilyl ester	899	SatFA_TMS	962.5 , 1.470	75	1758.6	821644
1200	Octanoic acid, trimethylsilyl ester	974	SatFA_TMS	1175 , 1.480	117	1731.2	442217
1511	Nonanoic acid, trimethylsilyl ester	988	SatFA_TMS	1387.5 , 1.475	117	9015.7	2393950
1808	Decanoic acid, trimethylsilyl ester	967	SatFA_TMS	1595 , 1.460	117	764.66	184704
2327	Dodecanoic acid, trimethylsilyl ester	929	SatFA_TMS	1980 , 1.445	117	1002	232718
2364	Ribitol, 1,2,3,4,5-pentakis-O-(trimethylsilyl)-	803	sugars_4TMS	2012.5 , 1.295	103	432.23	126318
2386	Ribitol, 1,2,3,4,5-pentakis-O-(trimethylsilyl)-	824	sugars_4TMS	2030 , 1.255	103	1652	426671
2397	Ribitol, 1,2,3,4,5-pentakis-O-(trimethylsilyl)-	831	sugars_4TMS	2040 , 1.265	103	5129.1	1670019
2772	Myristic acid, TMS derivative	807	SatFA_TMS	2330 , 1.440	285	509.48	83166
2775	3-Keto-glucose, bis(O-methyloxime), tetrakis-O-(trimethylsilyl)-	867	sugars_5TMS	2335 , 1.295	73	4663	1.2E+07
2914	D-Fructose, 1,3,4,5,6-pentakis-O-(trimethylsilyl)-, O-methyloxime	905	sugars_5TMS	2417.5 , 1.310	173	1584.1	1708356
2993	d-Mannose, 2,3,4,5,6-pentakis-O-(trimethylsilyl)-, o-methyloxyme, (1Z)-	925	sugars_5TMS	2445 , 1.300	73	8343.5	2.7E+07
3008	d-Galactose, 2,3,4,5,6-pentakis-O-(trimethylsilyl)-, o-methyloxyme, (1E)-	888	sugars_5TMS	2452.5 , 1.330	87	535.32	139884
3015	d-Glucose, 2,3,4,5,6-pentakis-O-(trimethylsilyl)-, o-methyloxyme, (1E)-	949	sugars_5TMS	2457.5 , 1.305	160	9738.5	1.2E+07
3027	Hexadecanoic acid, methyl ester	965	Linear_saturated_FAMES	2460 , 1.505	87	9592.1	3836100
3043	d-Mannose, 2,3,4,5,6-pentakis-O-(trimethylsilyl)-, o-methyloxyme, (1Z)-	938	sugars_5TMS	2467.5 , 0.235	205	1836.1	1537107
3099	d-Galactose, 2,3,4,5,6-pentakis-O-(trimethylsilyl)-, o-methyloxyme, (1Z)-	917	sugars_5TMS	2495 , 0.260	160	189.3	108498
3221	Ethyl pentadecanoate	909	FAEE	2570 , 1.495	88	1026.6	244166
3259	Palmitoleic acid 1TMS	822	MonoenoicFA_TMS	2612.5 , 1.450	117	169.84	53967
3266	15-Tetracosenoic acid, (Z)-, TMS derivative	869	MonoenoicFA_TMS	2620 , 1.455	117	342.54	132307
3293	Hexadecanoic acid, trimethylsilyl ester	970	SatFA_TMS	2650 , 1.445	132	15244	5192772
3362	9,12-Octadecadienoic acid, methyl ester	924	Linear_dienoicFAME	2730 , 1.520	67	4485.3	1692296

3371	9,12,15-Octadecatrienoic acid, methyl ester, (Z,Z,Z)-	939	Linear_trienoicFAME	2742.5 , 1.530	67	8735.7	3463550
3406	Octadecanoic acid, methyl ester	910	Linear_saturated_FAMES	2775 , 1.490	74	3421.7	1523706
3428	Heptadecanoic acid, TMS derivative	951	SatFA_TMS	2802.5 , 1.435	132	351.57	78943
3431	d-Galactose, 2,3,4,5,6-pentakis-O-(trimethylsilyl)-, o-methyloxyme, (1Z)-	896	sugars_5TMS	2807.5 , 1.320	73	10112	1.6E+07
3446	d-Galactose, 2,3,4,5,6-pentakis-O-(trimethylsilyl)-, o-methyloxyme, (1Z)-	908	sugars_5TMS	2830 , 1.325	103	1603.1	585452
3449	9,12-Octadecadienoic acid, methyl ester, (E,E)-	873	Linear_dienoicFAME	2832.5 , 1.520	67	447.44	161532
3456	d-Galactose, 2,3,4,5,6-pentakis-O-(trimethylsilyl)-, o-methyloxyme, (1Z)-	828	sugars_5TMS	2842.5 , 1.325	73	2108.3	2132816
3460	d-Galactose, 2,3,4,5,6-pentakis-O-(trimethylsilyl)-, o-methyloxyme, (1Z)-	814	sugars_5TMS	2850 , 1.345	147	713.49	235777
3464	d-Mannose, 2,3,4,5,6-pentakis-O-(trimethylsilyl)-, o-methyloxyme, (1Z)-	838	sugars_5TMS	2860 , 1.325	147	452.94	320633
3500	trans-9-Octadecenoic acid, trimethylsilyl ester	892	MonoenoicFA_TMS	2910 , 1.465	117	5742.2	944596
3503	α-Linolenic acid, trimethylsilyl ester	928	multienoicFA_TMS	2915 , 1.480	108	5737.4	1968665
3512	15-Tetracosenoic acid, (Z)-, TMS derivative	839	MonoenoicFA_TMS	2922.5 , 1.445	117	344.54	261762
3529	Octadecanoic acid, trimethylsilyl ester	988	SatFA_TMS	2945 , 1.450	117	13573	4039101
3620	Methyl (6E,9E,12E)-6,9,12-octadecatrienoate	623	FA	3047.5 , 1.560	91	98.991	31688
3780	Eicosanoic acid, trimethylsilyl ester	967	SatFA_TMS	3217.5 , 1.455	117	1436.2	310003
3900	Heneicosanoic acid, TMS derivative	814	SatFA_TMS	3345 , 1.475	132	103.32	15384
3964	Maltose 8TMS	833	sugars_8TMS	3425 , 1.375	204	10751	4491939
3982	15-Tetracosenoic acid, (Z)-, TMS derivative	825	MonoenoicFA_TMS	3442.5 , 1.500	117	225.79	94233
4012	Docosanoic acid, trimethylsilyl ester	880	SatFA_TMS	3467.5 , 1.495	132	487.28	55957
4159	Tricosanoic acid, trimethylsilyl ester	926	SatFA_TMS	3587.5 , 1.515	132	251.87	46322
4176	Maltose, octakis(trimethylsilyl)-	794	sugars_8TMS	3602.5 , 1.430	204	1589.4	771335
4193	D-Turanose, heptakis(trimethylsilyl)-	758	sugars_8TMS	3617.5 , 1.405	179	323.58	74146
4239	Maltose, octakis(trimethylsilyl)-	777	sugars_8TMS	3655 , 1.415	361	559.56	503216
4254	D-Turanose, heptakis(trimethylsilyl)-	840	sugars_8TMS	3665 , 1.440	73	3494.9	5710906
4300	Tetracosanoic acid, trimethylsilyl ester	965	SatFA_TMS	3702.5 , 1.545	117	1423.7	397263
4485	Maltose, octakis(trimethylsilyl)-	802	sugars_8TMS	3845 , 1.525	204	891.22	467784
4589	Docosanoic acid, trimethylsilyl ester	894	SatFA_TMS	3922.5 , 1.625	117	1394.4	447380
4718	3-Bromocholest-5-ene	770	FA	4032.5 , 1.785	105	90.826	22843
4810	Tetracosanoic acid, trimethylsilyl ester	804	SatFA_TMS	4127.5 , 1.755	117	1486.5	478749

4858	Campesterol tms	884	cholesterol_TMS	4192.5 , 1.860	129	4915.7	2095533
4965	β-Sitosterol trimethylsilyl ether	831	cholesterol_TMS	4282.5 , 1.965	129	8160.9	5024644
4981	Pregn-5-en-20-one, 3,16-bis[(trimethylsilyl)oxy]-, (3β,16α)-	742	sterol_TMS	4297.5 , 1.945	55	657.92	407467
5051	D-Turanose, heptakis(trimethylsilyl)-	848	sugars_8TMS	4367.5 , 1.935	73	2562.1	4776193

Fatty acid (FA); Fatty acid ethyl esters (FAEE); FAMES (fatty acid methyl esters); TMS (trimethylsilyl ether); SatFA_TMS (Saturated Fatty acid_trimethylsilyl ether).

Table S2. Twenty-nine compounds identified by SPME peaks that were detected by derivatization. ^{1}tR, ^{2}tR represent primary and secondary retention times, respectively.

Peak #	Name	Similarity	Classifications	1tR, 2tR (s)	UniqueMass	Quant S/N	Area
86	Hexanal	935	linear_aldehyde	602.5 , 2.010	41	1760.5	1278565
474	Hexanoic acid	951	linear_FA	1105 , 1.720	60	4149.4	2447456
486	Hexanoic acid, ethyl ester	932	FAEE	1112.5 , 1.835	88	805.48	154325
533	1-Isopropyl-4-methyl-1,4-cyclohexadiene	888	terpenes1	1160 , 1.430	93	4078.9	902565
586	4-Isopropenyl-1-methyl-1-cyclohexene	960	terpenes2	1192.5 , 1.405	68	34613	6260079
675	1,3,6-Octatriene, 3,7-dimethyl-	941	terpenes1	1240 , 1.430	93	18121	3095421
742	1-Isopropyl-4-methyl-1,4-cyclohexadiene	908	terpenes1	1272.5 , 1.395	93	5272.3	727499
847	1-Methyl-4-(1-methylethylidene)-1-cyclohexene	924	terpenes1	1352.5 , 1.375	93	10476	1795404
929	Nonanal	940	linear_aldehyde	1390 , 2.045	57	7715.1	1433560
1014	Octanoic acid, methyl ester	973	Linear_saturated_FAMES	1437.5 , 1.800	74	4037.3	1141873
1221	5,7-Octadien-2-ol, 2,6-dimethyl-	882	terpenes1	1545 , 1.785	93	357.1	74961
1455	Decanal	936	linear_aldehyde	1645 , 1.985	57	1976.9	496294
1566	Nonanoic acid, methyl ester	906	Linear_saturated_FAMES	1685 , 1.755	87	7480.9	1046417
1642	Bicyclo[2.2.1]hept-2-ene, 1,7,7-trimethyl-	839	terpenes1	1712.5 , 1.650	93	10292	1814198
2077	Decanoic acid, methyl ester	979	Linear_saturated_FAMES	1917.5 , 1.725	74	9841.9	2323317
2104	(1R,2S,4R)-2,7,7-Trimethylbicyclo[2.2.1]heptan-2-ol	733	terpenes2	1935 , 1.880	85	128.06	30844
2362	6-Dodecanone	855	ketones	2102.5 , 2.025	43	464.5	210649
2373	Dodecanal	945	linear_aldehyde	2110 , 1.890	41	1729.5	628829
2408	Undecanoic acid, methyl ester	935	Linear_saturated_FAMES	2140 , 1.705	74	505.85	122333
2535	Naphthalene, 1,2,3,5,6,7,8,8a-octahydro-1,8a-dimethyl-7-(1-methylethenyl)-, [1R-(1α,7β,8aα)]-	840	terpenes3	2245 , 1.505	91	717.6	134886
2631	Dodecanoic acid, methyl ester	944	Linear_saturated_FAMES	2347.5 , 1.670	74	10186	1875553
2802	Patchouli alcohol	850	terpenes3	2655 , 1.740	41	132.78	42116
2835	Tetradecanoic acid, methyl ester	969	Linear_saturated_FAMES	2732.5 , 1.625	74	2363.3	643488

2890	Pentadecanoic acid, methyl ester	960	Linear_saturated_FAMES	2877.5 , 1.245	74	587.4	83335
2904	2-Undecanone, 6,10-dimethyl-	868	ketones	2900 , 1.320	43	651.19	145010
2941	Hexadecanoic acid, methyl ester	990	Linear_saturated_FAMES	2970 , 1.155	74	48039	6751820
3022	9,12-Octadecadienoic acid, methyl ester, (E,E)-	912	Linear_dienoicFAME	3085 , 1.110	67	1066.3	142572
3025	9,12,15-Octadecatrienoic acid, methyl ester, (Z,Z,Z)-	908	terpenes3	3090 , 1.110	79	858.09	184721
3032	Tetradecanoic acid, methyl ester	889	Linear_saturated_FAMES	3100 , 1.100	74	621	94072

Fatty acid (FA); Fatty acid ethyl esters (FAEE); FAMES (fatty acid methyl esters).

4. TLC plate of all fraction of *Acalypha arvensis* Poepp

Figure S9. **TLC plate of all fraction of *Acalypha arvensis* Poepp**

HPTLC plates of the six fractions obtained from *A. arvensis*. Fractions were prepared at a concentration of 5mg/mL and deposited on a silica gel 60 F254 20 × 10 cm HPTLC plate (Merck) using the CAMAG Automatic TLC Sampler 4 (ATS 4) depositor (CAMAG, Muttenz, Switzerland). A deposit of 5 µL was thus carried out in three replicates for each fraction. The migration was performed with the eluent system ethyl acetate/methanol (90/10) over a distance of 70 mm with the CAMAG Automatic Developing Chamber 2 (ADC 2). The developed TLC plates were visualized under Visible light (A), UV 254 (B) and 366 nm (C) using the CAMAG TLC Visualizer.

(A)

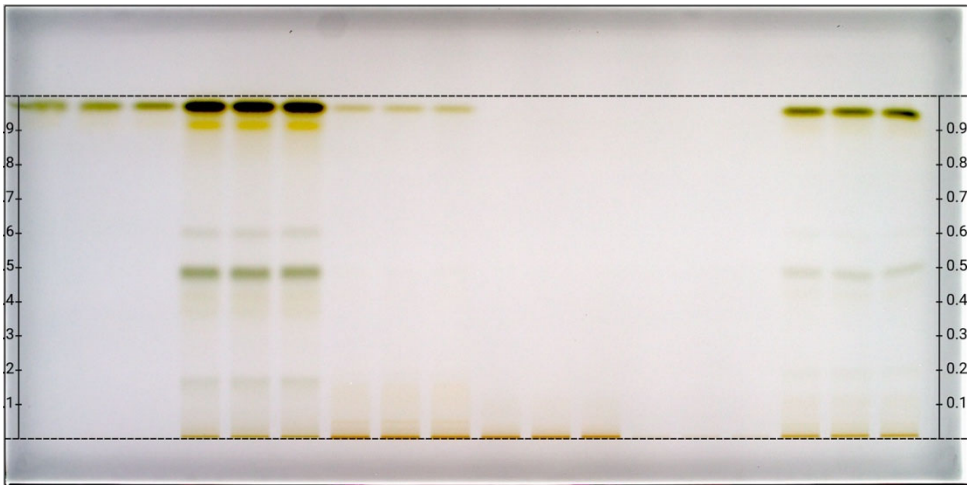

F1 F2 F3 F4 F5 F6

(B)

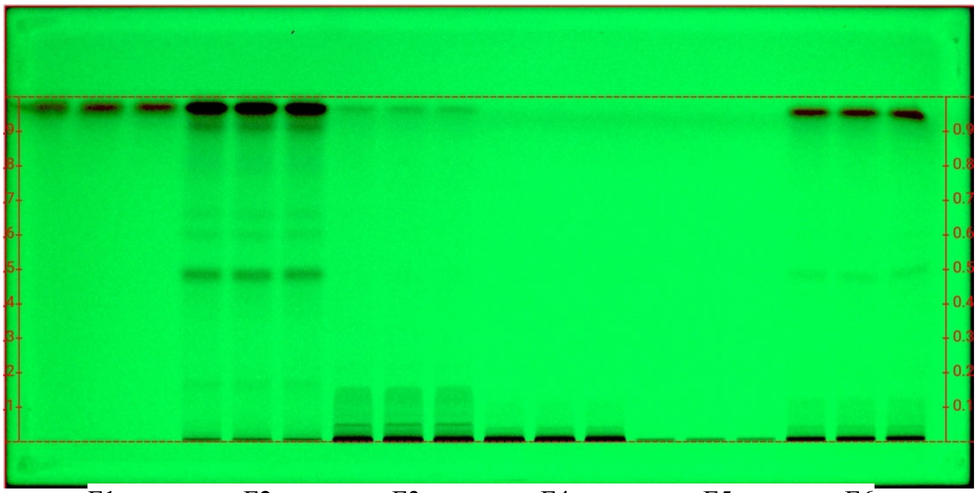

F1 F2 F3 F4 F5 F6

(C)

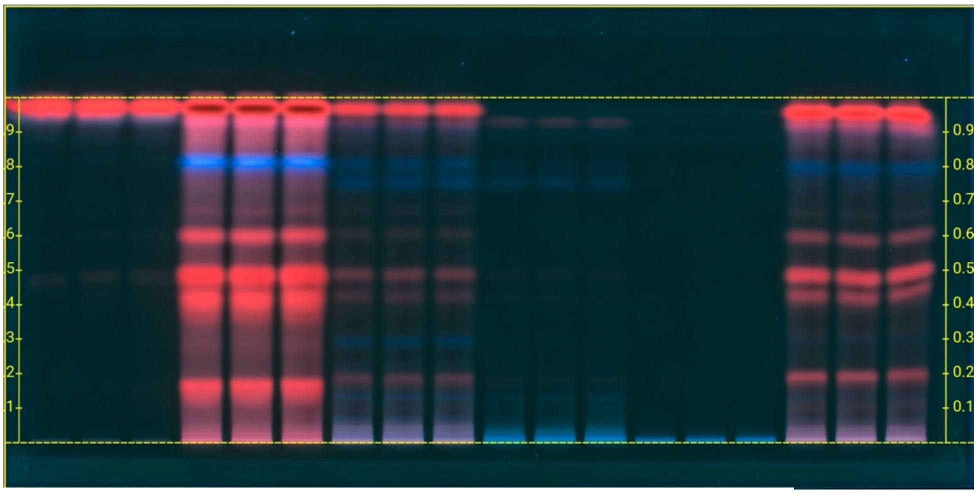

F1 F2 F3 F4 F5 F6
